# Postgraduate Medical Education in the South West

**Published:** 1979

**Authors:** J. E. Cates

**Affiliations:** Postgraduate Medical Dean, University of Bristol Emeritus Physician to the Avon Area Health Authority (Teaching)


### Postgraduate Medical Education in the South West

**J. E. Cates**
Postgraduate Medical Dean, University of Bristol
Emeritus Physician to the Avon Area Health Authority (Teaching)
*Long Fox Lecture, delivered November, 1979*

Over a century ago, as a Lecturer in Medicine and Pathological Anatomy, Edward Long Fox had sound ideas about medical education in advance of his time. He took a personal interest in his pupils, guiding their studies, frequently asking them to his home, and he sometimes gave them standard works on medicine. He himself tried to keep up-to-date, even reading books and journals whilst in his carriage going on his rounds. He helped to raise funds from other doctors to provide a medical library and considered that this and a separate room for medical meetings should be connected with the University College.[1] It is appropriate that this Long Fox Memorial Lecture should review medical education of doctors in this Region.

**HISTORICAL BACKGROUND**

In his Rock Carling Fellowship, Professor Robert Milnes Walker tells us how medical education evolved through the centuries.[2] Henry VIII passed a law in 1511 which laid down that 'no person within the City of London, nor within seven miles of the same take upon him to exercise and occupy as a Physician or Surgeon, except he be first examined, approved and admitted by the Bishop of London, or by the Dean of St. Paul's for the time being'.

At our end of history Milnes Walker suggested that the pre-registration training should be for two years rather than one — an idea more forcibly argued in the Merrison Report, 1975.[3]

Milnes Walker points to two cardinal events: firstly the Haldane Commission in 1913 recommended that there should be full-time clinical units in London teaching hospitals. Only a few professorial departments were established between the wars, yet their influence is now immense. Students learn how to put dogma and empiricism on trial; housemen and registrars move sideways into the NHS and take with them some of the high standards of clinical science they learnt on a unit, and weave these into better patient care. Scientific method and research both advance knowledge and improve medical service to patients.

The second event was the publication of that wise, far-sighted work, the Goodenough Report — or the 'Report of the Inter-departmental Committee on Medical Schools' (1944) — which was adopted by the government of the day.[4] This was the most important step forward in medical education in this century. It was concerned mostly with undergraduate education and the need for a pre-registration year. Of postgraduate medical education it said:

> 'Properly planned and carefully conducted medical education is *the* essential foundation of a comprehensive health service. If such a service is to have continuing vitality it must be founded on highly developed and vigorous systems of general and professional education for members of the medical and allied professions, and it must evoke the enthusiastic and intelligent co-operation of the general public.
>
> 'The spirit of education must permeate the whole of the health service, and that service must be so designed and conducted that, among other things, it secures for medical education the necessary staff, accommodation, equipment and facilities. Medical education cannot be regarded as merely incidental to the hospital service.
>
> 'Medical education and research are of vital importance to the nation, and the general public should maintain a lively and understanding interest in them.
>
> 'Indeed, the nature and results of the arrangements made for training medical practitioners, and for increasing their knowledge and skill after qualification, can be rightly judged only in relation to the individual and collective needs and well-being of the public.
>
> 'Each university having a medical faculty should play its part, not as a rule by providing courses in its undergraduate teaching centre, but by arranging courses in suitable hospitals which are in the zone of influence of the university.
>
> 'Each university should appoint a special committee or board of postgraduate medical studies and should also depute a person to undertake the organisation and general supervision.'

Thus the broad structure of medical postgraduate education today was designed during the dark days of World War II.

The Goodenough Committee also advised the government of the day that universities should organise courses for doctors demobilised from the forces. The University of Bristol was ready for these ex-service doctors. Mr. Jack Wright was appointed Director of Postgraduate Studies, and he chaired the Medical Postgraduate Committee. The first meeting was on 11th October 1945 and Professor Bruce Perry attended that meeting and most of the later ones. Many supernumerary registrar posts were created and several retraining programmes were set up.

In 1946 the University established another body, the Postgraduate Advisory Council, with delegates from all of the South West. The then Vice-Chancellor, Sir Philip Morris, concerned himself closely with this Council. He was fond of saying a University was not just a building where students become graduates. A University, he insisted, should have a zone of influence — and our influence should extend from Penzance to Cheltenham, For years this Council was the forum where progress in medical postgraduate education was kept under review, and the day to day business was planned by the Medical Postgraduate Committee.

As the functions of medical postgraduate education became more complicated, its administration had to be separated into several smaller bodies and committees. The Council began to have a less clear use and was disbanded in 1973. Its minutes each year provide a good record of all that was achieved in that period.

On 1st August 1950 Dr. Arthur Gale succeeded Mr. Jack Wright as Director of Medical Postgraduate Studies. His office was a small, bare, stone-faced room in the basement of the tower of the Wills Memorial Building, which had been used by the Bristol Medico-Chirurgical Society.

At Dr. Gale's first meeting it was recommended that Registrars should rotate between the teaching hospital and Regional Hospital Board hospitals. A year later it was proposed that RHB hospitals should appoint 'postgraduate supervisors' to look after pre-registration housemen, when they were eventually appointed.

The minutes of meetings of those days recorded the setting up of temporary consultant posts at £1,000 a year, and providing courses in Bristol to prepare for Diplomas in Child Health, Mental Health, Public Health and so on. There are lists of those consultants selected to give lectures in the main hospitals throughout the region.

Dr. Gale died on 12th October 1956. His initiative and influence are yearly commemorated in the Gale Memorial Lecture promoted by the South West Faculty of the Royal College of General Practitioners. The Dean, Professor Tom Hewer, took over his duties for a few months. In 1957 I was invited to be Medical Postgraduate Dean and was duly appointed by Senate.

**EFFECT OF THE HEALTH ACT**

The National Health Service Act of 1946 came into effect in 1948. Under Section 48 of that Act the Department of Health promoted the continuing education of general medical practitioners.[5] The Department of Health deputed to universities the responsibility of arranging and advertising courses and this was done by postgraduate deans and the postgraduate committees. Lecturers' fees were refunded by the Department of Health and doctors who attended could claim back their expenses. In 1968, in the Health Services and Public Health Act, Section 63 continued these arrangements.

Here is an essential principle: the DHSS accepts that it is responsible for professional training of doctors, and asks universities to arrange this education. In practice, universities have depended on NHS consultants to do most of this teaching.

In the Todd Report[6] this principle was re-inforced. Preparation for higher degrees is a proper object for the expenditure of university funds; training of a more professional, rather than academic character ought to be paid for by the National Health Service. In 1973 the DHSS, the UGC and the Committee of Vice-Chancellors and Principals accepted this principle and defined their respective financial roles in Postgraduate Medical Education.[7]

Besides establishing this basic principle, Section 48 had a catalytic effect. Universities arranged courses and designated selected consultants in peripheral hospitals to help with organising this work. In this region Dr. R. G. Anderson (Cheltenham), Dr. Charles Andrews, Dr. Dennis Hardy (Truro), Dr. Lovell Hoffman (Bath), Dr. Richard Jarrett (Gloucester), Dr. Francis Page (Southmead), Mr. W. B. Waterfall (Plymouth) and Dr. Brian Webb (Taunton), were among these pioneers over 20 years ago. These consultants were the forerunners of university appointed clinical tutors.

**THE CHRIST CHURCH CONFERENCE AND THE NUFFIELD PROVINCIAL HOSPITALS TRUST**

In December 1961 the Nuffield Provincial Hospitals Trust convened a conference in Christ Church, Oxford[8] chaired by Sir George Pickering.

Representatives from the main bodies concerned in medical postgraduate education attended. The recommendations were strong, clear and simple:

1. Broadly it was agreed that medical education was a continuing process.
2. All branches of the medical profession should have access to a library and to discussion groups.
3. All posts in hospitals from pre-registration to senior registrar were to be recognised as training posts.
4. The basic Regional Postgraduate Training Unit should be the district (general) hospital.
5. All consultants should recognise that training of their junior staff was one of the most important aspects of their work.
6. Senior and junior staff should have time to devote to this. It was important to promote an educational atmosphere.
7. In each of these hospitals a consultant should be nominated by the Regional Committee for Postgraduate Education as Clinical Tutor, with administrative support and secretarial assistance. He should see that general practitioners had access to the library and participate in clinicopathological conferences.
8. There should be certain 'physical facilities' including a seminar room, a medical library with a part-time librarian, clinical tutor's room and a lunch room as a focal point where hospital medical staff could meet and be joined by general practitioners.
9. There should be a Postgraduate Dean and a strong Regional Committee for Postgraduate Education.

In our region, due to the wisdom of our then Vice-Chancellor, the bones of much of the Christ Church recommendations were already there. It was easy to give the title of Clinical Tutor to those who were already doing the work. But libraries and secretaries needed money.

We were all thrilled that after the conference the NPHT set aside £250,000 spread over five years to fund medical postgraduate education in the provinces. We received a fair share of this: £18,500 spread over three years. The Nuffield made further grants toward building centres: Exeter £30,000 and Plymouth £8,000.

**POSTGRADUATE MEDICAL CENTRES**

First let's look at what the Christ Church Conference called 'physical facilities' and how this need has been met by providing postgraduate centres.

At the time of the Christ Church Conference only a few Regional Board Hospitals had a good medical library or a conference room; those that had owed them not to the NHS but to local medical societies. In our region Exeter and Bath were fortunate for this reason. In Bristol the library of the Medico-Chirurgical Society had been combined with that of the University and so Medico- Chirurgical members enjoy the freedom to use the library.

In the main, however, the 'physical facilities' were poor, and there were no NHS funds for building centres. So groups of doctors all over the country began making their own plans and launched appeals for the necessary money. This has been called the Postgraduate Centre Movement.[9] John Lister has recorded the centres opened each year since 1962. The last count in September 1979,[10] is 339 centres in England and Wales. Each year the Council for Postgraduate Medical Education publishes a directory of all centres in England and Wales.[11]

To start with, funds were raised by appeals to our profession — hospital staff and local practitioners, to industry including the pharmaceutical houses, trusts and charities: the earlier centres in this region were built almost entirely from money collected in this way.

In 1967 the NHS applauded the success of postgraduate centres and advised that each district general hospital should have its own centre:[12]

> 'The main purpose of postgraduate centres is to provide for the needs for continuing education of doctors in permanent appointments and in general practice and for the vocational training of those doctors occupying training posts.' Centres should help with retraining of married women doctors. 'It seems desirable . . . to widen the scope of these centres to assist in the continuing education of dentists, pharmacists, nurses and professions supplementary to medicine. The activities of a centre may extend to research projects . . . particularly . . . in community health.'

In the following year (1968) the Ministry circulated a 'Design Guide' for postgraduate medical centres giving two alternatives:

(a) plans for a postgraduate medical centre
(b) plans for a hospital education centre to cover training of nurses and allied professions as well as doctors.

Naturally, if the NHS authorities were making plans to pay for and build a 'larger hospital education centre' then it would be likely that doctors would find themselves competing with, say, the sister tutor for use of lecture theatres. This conflict came to a head with headlines of 'Hands off Postgraduate Centres'.[13] There was then an amicable confrontation between Sir George Godber on the one hand and Clinical Tutors and Deans on the other. The outcome was that the Postgraduate Medical Centre Group was established, and after several meetings the Group produced a report setting out the desired provisions of a postgraduate medical centre. The Council for Postgraduate Medical Education published this in 1974.[14]

Ideally a postgraduate medical centre should be purpose built. If there were no funds for this ideal, then it had to be a multi-disciplinary centre. But in such a multi-purpose centre there must be a clearly identifiable medical component over which the medical tutor has authority. In the building of new centres this advice is not always heeded. Likewise the Ministry require those planning new centres to have 'prior consultation' with universities, postgraduate deans and many others concerned, but the planners have sometimes 'gone it alone'.

Recently some new centres have been built in Wales and the Home Counties using such design pieces as Harness, Best Buy, or Nucleus. Where the accommodation has been designed to be shared the results have not been a complete success.

So the struggle continues. The DHSS agreed to re-form the Postgraduate Medical Centre Group to re-define the required facilities, and recommendations are well advanced.

In this region, since 1968, eleven *major* centres have been completed; most of these were started as the result of local initiative and effort. Tutors and their colleagues in our District General Hospitals and those in general practices deserve credit for these fine buildings and for the lively activities which continue in them. The Table shows some details of these centres. Money for the earlier centres came from local appeals. The total cost of these centres is £753,000; of this total £240,000 (a third) was raised by appeals. Excluding the UBH, and Bath after joining Wessex, we are left with centres in our District General Hospitals and about a half of the cost was met by

**MAIN POSTGRADUATE CENTRES IN THE SOUTH WESTERN REGION**

| *Place* | *Date Opened* | *Cost* £ | *Money Subscribed* £ | *Comments* |
|---|---|---|---|---|
| Bath | 1968 | 40,000 | 20,000 | |
| Extension | | 140,000 | 20,000 | (now Wessex) |
| Torquay | Nov. 1968 | 42,000 | 42,000 | Funds growing |
| Taunton | Mar. 1969 | 35,000 | 20,000 | |
| Plymouth | April 1969 | 42,000 | 42,000 | |
| Extension | 1975 | 16,000 | 4,000 | |
| Exeter | Oct. 1970 | 140,000 | 20,000 | Northcott Foundation |
| Car Park | | 9,000 | 12,000 | Dorothy McKenzie Trust |
| | | | 15,000 | Appeal |
| Cheltenham | Oct. 1971 | 8,700 | 4,000 | |
| Frenchay | Nov. 1972 | 60,000 | 35,000 | |
| Yeovil | Feb. 1974 | 75,000 | – | |
| Weston | Sept. 1975 | 11,500 | – | |
| Gloucester | June 1976 | 34,500 | 6,000 | |
| North Devon | Mar. 1978 | ? | 20,000 | Too small |
| UBH (Edward Jenner Centre) | 1977 | 100,000 | – | Shared with students |
| *Projected* | | | | |
| Southmead | Summer 1981 (Construction in progress) | 250,000 | 22,500 | Functionally integrated with UGC/RHB undergraduate unit |
| Treliske | | 300,000 | | |
| Weston-super-Mare | | at planning stage for new hospital | | |

Minor conversions have been made in Winford and Ham Green etc.

funds raised from appeals. In replying to a recent survey our tutors in this region felt that most centres, though built 10 years ago, provide enough lecture space; the chief need was for more seminar rooms. However, if postgraduate medical education was to continue expanding and was to embrace 'professions supplementary to medicine', centres would have to be enlarged.

There are several lessons we have learnt.

1. A schedule of accommodation and physical facilities has to be agreed at top level. A modern postgraduate centre should have prescribed lecture rooms, library, seminar and discussion rooms, common room, catering and bar facilities, cloakrooms, toilets, offices for tutors' secretaries and car parking.
2. As soon as there is word of a new hospital, even before plans are drawn up, everyone should help to fashion as good a postgraduate centre as they can.
3. In designing a medical centre one must have an eye for the future and allow for expansion.
4. Design of a centre should be left to those who know local needs and peculiarities. Harness, Best Buy and Nucleus are made to fit into the template and cannot be adopted for tailor-made centres.
5. Even if the Health Authorities are prepared to meet all the cost, there should still be an appeal for the centre. This helps in two ways:
   (a) with such a fund doctors may ensure alterations and improvements in furnishing or design
   (b) the appeal whets local interest and enthusiasm: a centre you have helped to pay for is a centre you wish to see flourish later. You have a pride in the place.

Amongst the many who have struggled hard to achieve so much are Drs. Hugh Leather, Brian Webb, Francis Page, John Zorab, Tony Bowyer, Mr. Reggie Merryweather and Professor Alan Read. All are, or have been, clinical tutors.

**CLINICAL TUTORS**

In 1964 the Department of Health agreed to take over from the NPHT the funding of honoraria for tutors, their appointment and actual payment remaining in the hands of universities.

The duties of the University-appointed Clinical Tutors have become more complex and exacting. The Tutor is in overall charge of the postgraduate medical centre. He should have a secretary and librarian. There will be a district postgraduate committee (or medical centre committee) which the Tutor chairs. On this committee there will be: (i) some hospital colleagues, perhaps chairmen of the 'cogwheel' divisions, (ii) a junior hospital doctor, (iii) a dentist, (iv) representatives from other hospitals — geriatrician, psychiatrist, etc., (v) community health doctor. There may be tutors from faculties and colleges — and one or two general practitioners.

With the help of this committee the tutor will plan the various programmes:

(i) For general practitioners: Lunch-time meetings, study days, a week's continuous course, extended courses. Most of these will be in the centre though sometimes the meetings take place in health centres round about.

(ii) Trainees for general practice: Once a week there are 'Half-day Release' courses for trainees in general practice. These are attended by trainees doing their year in a practice and those doing their two years in hospital posts as part of a vocational training scheme. Trainers attend these courses too. Details of these courses are in the hands of the general practitioners appointed as course organisers; the Tutor will be concerned with these activities and provide space for the organisers, for the lectures, and maybe secretarial help.

(iii) There will be activities to arrange for the hospital doctors: the regular staff rounds, grand rounds, clinicopathological conferences, journal clubs, etc. Tutorials will need arranging for junior hospital doctors aspiring to higher degrees, and there are lectures from outside speakers to promote.

Though much teaching is still done for love, the more formal teaching can earn the lecturer a fee; and, so that those who attend may claim expenses, an attendance list may be needed.

The methods of payment are different and the paperwork necessarily tedious. Those courses for general practitioners and trainees are under 'Section 63', the money coming from the DHSS through the University Finance Office. Lectures by hospital doctors to hospital doctors are paid for by the Regional Health Authority.[15] For some courses an attendance fee has to be collected by the Tutor; and this fee can be reclaimed from the employing authorities if the course has been approved by the Regional Postgraduate Medical Education Committee under HM(67)27.

The Tutor is sometimes helped by a Medical Library Committee, to choose books and journals. Hospitals are visited by teams to decide whether posts are of a standard needed for training, be they pre-registration, SHO, registrar or senior registrar posts in the wards, in the laboratories, Departments of Radiology, and so on. These visitors expect the centre's medical library to be well stocked with journals and books related to their subject, and will enquire about programmes of lectures and seminars for junior hospital doctors in their particular specialty.

GPs may come to the Tutor seeking individual attachment to one of the clinical departments, to ask him for help about obtaining prolonged study leave for, say, three months to work in a practice overseas or sit for a higher exam.

Married women doctors (Doctors with Domestic Committments) come to ask about the Retainer Scheme which just keeps them in touch with medicine. The Clinical Tutors have been charged with providing them with advice and guidance and will supervise their education sessions. In their later retraining and search for part-time posts, 'DDCs' may again turn to the Tutor for help.[16] Tutors are grateful, as are the rest of us in this region, to Dr. Beryl Corner for the ever-ready help and counselling she gives to DDCs and other women doctors.

Career guidance for other doctors is to be provided in part by Clinical Tutors (though the doctor's own chief is more responsible here, and the colleges' Regional Advisers and Postgraduate Deans have a role too). A section in medical libraries in all centres should be stocked with information sheets and booklets about career prospects and training requirements.

In a few centres you will find a 'centre manager'. However, in most centres the Tutor has to see to most of the managing. There is the masterminding of all the teaching and learning and publishing programmes and being host to lecturers. The Tutor must be *au fait* with audio-visual aids and those films which have been approved by the Leeds panel as free from promotional bias. There are guide-lines on accepting grants and sponsorship from drug firms without obtrusive advertising. Licensing laws for control of the bar, fire insurance, the need for an inventory of the centre's contents, the pros and cons of charity status.

Tutors have to confer with each other, and serve on various committees. At the Regional Postgraduate Medical Education Committee they discuss ways to meet needs — a course in obstetrics this year, one for trainers in general practice, preparation of junior hospital doctors for higher diplomas. They have formed their own committees and working parties to set standards for medical libraries and so on.

In 1968 the University Clinical Tutors formed themselves into a national association with the blessing of all concerned. They meet twice a year and their deliberations are attended by eminent folk such as the Chief Medical Officer. Tutors have representatives on the Council for Postgraduate Medical Education, and the UK Conference of Deans. We are proud of the role played by our Tutors in the Association, particularly Dr. Leather who has been their President and Dr. Bowyer who is now their Secretary and chairs their working party on medical library and audio-visual aids.

There is, however, a limit to what a Clinical Tutor will do. The General Medical Council depute to universities the task of deciding which house officer posts should be recognised for pre-registration training. After the Conference in 1973, medical schools and universities were required under the 'Code of Good Practice' to invite feedback from their graduates about their posts (as well as news of how the graduates fared from the consultants for whom they worked). Sometimes graduates have grounds for complaints; the university-appointed Tutors seemed just the men to help universities to keep in the picture. But this job they stoutly refused; they would not act as informants on their clinical colleagues.

One may marvel that any hospital consultants could be willing to tackle all these tasks, yet we have never lacked volunteers. In this region we have a Regional Health Authority anxious to promote medical postgraduate education with a warm hearted, well motivated servant of the authority — our Regional Postgraduate co-ordinator Dr. C. W. Davies. The Authority has found money to give the University to pay the honoraria for two and sometimes three Tutors per centre. In larger centres with three Tutors, the University tries to appoint a general practitioner who is prepared to share in taking on part of these duties. This University is the only one to invite selected general practitioners to become university-appointed tutors in postgraduate centres.

Some may have doubts about the value of all this education and training. Sir Francis Avery Jones (1968) has however described how a good district general hospital can develop undergraduate and postgraduate teaching and research to compliment the work of the main teaching hospitals. He stresses that 'teaching and research go hand in hand because both are concerned with advance of medical knowledge'. Those hospitals which have been able to provide good facilities for teaching and research have had the best applicants for posts (both junior and senior medical staff). This is reflected in a rise in the standard of patient-care and it pays good dividends in lowered mortality. This applies most to life-threatening emergencies when there are keen well-trained junior doctors. The appointment of a clinical tutor has had major impact on the organisation of teaching.

**TRAINING COMMITTEES**

Training of junior hospital doctors from pre-registration house officers to senior registrar is essentially apprenticeship. It is the oldest system for learning, and it is still the best. You need only to read obituaries for, say, R. V. Cooke by those who learnt from him to realize what good teachers our best chiefs have been. However, it is widely agreed that this apprenticeship system needs some cohesion and integration because the required experience is diverse, for example in anaesthetics, and experience has to be gained in many specialised skills. As a result of the Report of the Working Party on Medical Staffing Structure in Hospital (HMSO 1961) the NHS in HM(61)119 required each Regional Hospital Board to establish a Regional Joint Advisory Committee on Senior Registrars.

The committee should:

(i) supervise, assist and advise on the careers of senior registrars in the region;
(ii) advise on the organisation and development of rotational training schemes;
(iii) consider reports on all senior registrars who are at the end of their first year of training;
(iv) consider and advise the best course to be taken where a first year senior registrar has an unfavourable report and where a senior registrar fails to secure a consultant appointment after five years in the grade.

In our Region this committee was chaired for many years by Dr. Stewart Smith. This group earned the respect of consultants and the senior registrars who worked for them; they were a jump ahead of all the Joint Committees of Higher Training because they concerned themselves with the educational standards of the posts: they asked whether a senior registrar had time each week for study and research, or whether he had been granted his study leave, and whether he was happy that his experience was varied enough.

The work of this RJAC on Senior Registrars however, was done by a few old faithfuls — and when the Health Service was re-organised Dr. Duncan Egdell (who served for many years as postgraduate co-ordinator while holding his post with the Regional Health Authority) devised our present Regional Committee for Specialist Training. The main committee has been chaired with patience, tact and skill by Mr. Herbert Bourns for three years: there are five sub-committees, medicine, surgery, etc. and these consist largely of consultants in the appropriate subject including the College Adviser and University Head of Department. These sub-committees are strong and authoritative, and could well take over from the visiting teams of the Higher Training Committees the role of approving posts and training programmes with, perhaps, one co-opted from a neighbouring region. This would save time and money.

**UNIVERSITY**

The role of universities in all postgraduate education is changing. Other professional people, such as engineers, are recognising that learning does not stop at graduation and that universities should accept this extension of their educational duties.

In the University of Bristol we now have Chairs in most clinical disciplines — medicine, surgery, child health, radiology, mental health, obstetrics and gynaecology, anaesthetics, microbiology, and others are being planned; and there are lecturers in charge of other clinical departments. All these contribute to postgraduate education of junior hospital doctors and general practitioners. Most clinical and preclinical departments now mount courses to prepare junior hospital doctors for examinations of the Royal Colleges — Medicine, Surgery, Pathology, Psychiatry, and Anaesthetics. All departments contribute to courses for general practitioners and some go to much trouble: Professor Read takes members of his team on a peregrine tour to centres miles away; Mr. John Farrell in dentistry has done the same.

This role of the University in its zone of influence is sure to increase. In our region Exeter, a University without a full Faculty of Medicine, established a Department of Postgraduate Medicine in 1964. David Mattingly, the director, and his colleagues have built up courses of postgraduate education aimed at overseas graduates and those from this country who wish to retrain. These courses last 10 weeks each term. Exeter University established a Department of General Practice headed by Dr. Denis Pereira Gray. Both these ventures have been most successful and the University of Bristol is gratified that it lent all its support to the launching of both projects.

We should acknowledge all that NHS consultants do in return in postgraduate medical education; they take our students for electives, and train them when pre-registration house officers, and so on. Is it not time for them to receive some accolade of university status to recognise their contributions in this field? After all, general practice course organisers are 'recognised teachers in general practice' within the University.

### TEACHING IN GENERAL PRACTICE

For many years our undergraduates have been attached to general practices at the beginning of their clinical course and again in the final year. They accompany selected general practitioners in both town and country practices with whom they live for a fortnight. They receive lectures too from GPs and others, on topics dealt with by practitioners.

### VOCATIONAL TRAINING FOR GENERAL PRACTICE

An experimental scheme for vocational training in general practice began in Bristol fifteen years ago.[1 7] Two sets of four posts were created in which the holders had four posts of three months each in various hospital specialties, and then undertook traineeships in general practice. Again, the NPHT gave us a grant, this time to make up the difference in salaries of the trainees. Though this scheme collapsed, we learnt from it, and informed the Todd Commissioners of our experiences and conclusion that vocational training schemes for general practice should be made mandatory. This is a recommendation that finds its place in the Royal Commission's Report (in paragraph 117).[6]

By 1970, vocational schemes had been started in London and seven regions; so we began again. We appointed an 'organiser' (a forerunner of the Regional Advisers) and NPHT gave a grant of £1,000 to cover the cost of this post. Dr. Michael Lennard was appointed and has dedicated himself to the cause of better training for general practitioners ever since. This time, the formidable task of constructing rotations of selected hospital posts was entrusted to a working party: Drs. Ken Arney, Terry Beddoe, Michael Lennard and Donald Ractliffe. Dr. Ractliffe chaired this team regularly until July 1977 under the guidance of the Regional General Practice Sub-Committee.

Other rotations have been established in all the larger centres in our region. Today there are 195 trainees for general practice in this region. There are 120 in constructed schemes and 75 are making their own choice of jobs. The training lasts 3 years, so the yearly output of doctors trained to be principals in general practice is 65 — the target is 75 by the end of 1980. Doctors who apply for a place in these schemes are of a high standard. Each six months there are 5 vacancies in the Bristol scheme and there are 30 applicants or more. Shortly every hospital post that trainees might work in will have to be assessed for approval for general practice training. More GP trainers are needed, and these trainers will need to learn about how to train.

Sir George Pickering[9] said 'what has been done in the name of postgraduate education since the war has consisted of lecture and refresher courses. These are alright in their way, but they are not of much account. Indeed, they have performed a disservice in providing a facade behind which is emptiness. Education programmes at postgraduate level should largely consist of discussions which may be centred on patients or ideas, or new work'.

The Royal College of General Practitioners has done much to promote progress in education; seminars, workshops, audits, peer reviews are now widely used methods of learning in GP vocational training schemes. Advisers have a special remit of trying to assess the value of these different teaching and learning activities. Trainers are encouraged to use heuristic and Socratic methods rather than didactic teaching; and trainees are stimulated to undertake research projects during their general practice trainee year.

### THE FUTURE

This is not meant to be a self-satisfied review. The Report of the Royal Commission on the National Health Service tells us that more needs to be done to raise standards of medical care and provide a better health service.[18] The Commission agrees with the Royal College of General Practitioners that the care provided by some doctors in general practice is mediocre, and that provided by a minority is of an unacceptably low standard.

The Commission says this of education of students: 'The undergraduate education of doctors gives relatively little emphasis to experience in the community'. This is similar to the charge levelled thirty-five years ago in the Goodenough Report,[4] which asserted that in the training of medical students: 'more attention must be given to minor ailments, the more common diseases and the early stages of disease, to chronic diseases, to infections, to rehabilitation'. (p.28(4)). The Commission states that the 'medical student should be far better prepared than he is at present for team working with other disciplines'. (17.29). So the education of students needs to be more relevant to the problems met in general practice and primary care.

Of the education of doctors the Commission says that though compulsory vocational training will help to raise standards 'general practitioner training is often inadequate'. The Royal Commission goes on: 'Medical education should be relevant to the major health problems of the day and amongst these are now geriatric illness, mental illness, disability and handicaps and the potentially preventable diseases and injuries which result from an unhealthy life-style'.

'There should be more emphasis on community care . . .' (17, 29–30).

Our Regional Postgraduate Committee has pointed to ENT, orthopaedics and ophthalmology as specialties where general practitioners feel the need of further training. The out-patient waiting lists are prolonged by requests for consultant opinion on minor problems. Clinical attachment to out-patient clinics could give GP experience and confidence in these fields.

The Commission recommends that one way to raise standards would be more *research* in general practice and primary care, and it asserts 'The general practitioner is ideally placed to study the natural history of disease, the family setting of illness and the recurrent or chronic conditions'. (7,34).

The Commission hammers again at the need for research. 'Research is vital to improve standards of patient care. It increases knowledge and fosters a critical attitude to existing patterns of care and treatment (17,34) . . . Clinical research should be accepted as part of postgraduate education.' (17,36).

All these quotations are enough to make us embarrassed by our shortcomings. What can be done to give students and doctors a better education and provide facilities and opportunities for research?

We have already acclaimed the immense benefits to patient care that have come from establishing full-time university departments in the main clinical disciplines. A discipline may be recognised by its having its own body of knowledge, its skills and its attitudes: it can be argued that general practice has every right to claim that it is as much a discipline as any other branch of the medical profession. Surely the best way, the only way, to promote the improvements in undergraduate and postgraduate teaching and to stimulate research is to have a lively University Department of General Practice. The staff will have to have proven ability in research and teaching. They all need time for the job, and proper facilities. Such a department should advance knowledge, improve standards and inspire others to do the same.

The Commission goes on 'the universities have been criticised for being slow to develop academic departments in fields which have been chosen by health departments as deserving priority' (17,31) and 'we recommend that the health departments should, as a matter of national policy fund chairs or promote joint University/NHS appointments . . . in priority specialties'.

There are now established departments of General Practice in all Universities with medical faculties — except for Bristol, so we are the last.

Last century, Edward Long Fox had high standards and ideas on education in advance of his time, and did his best to put these into effect. If he were here today would he not agree with those of us who feel that if we are the last to have a University Department of General Practice, then we must make sure that it is the best?

